# Global map and indicators of food system sustainability

**DOI:** 10.1038/s41597-019-0301-5

**Published:** 2019-11-25

**Authors:** Christophe Béné, Steven D. Prager, Harold A. E. Achicanoy, Patricia Alvarez Toro, Lea Lamotte, Camila Bonilla, Brendan R. Mapes

**Affiliations:** 1 Decision and Policy Analysis Program, International Center for Tropical Agriculture, Km 17 Recta Cali-Palmira, CP 763537 Cali, Colombia; 2 Supagro, 2 place Pierre Viala, 34060 Montpellier, France; 3 Department of Environmental Science and Policy, University of California, One Shields Avenue, Davis, USA; 4 Frederick S. Pardee Center for International Futures, University of Denver, 2201 South Gaylord Street, Denver, CO 80208-0500 USA

**Keywords:** Agriculture, Social sciences

## Abstract

This paper presents the first global map of food systems sustainability based on a rigorous protocol. The choice of the metric dimensions, as well as the individual indicators included in the metric, were initially identified from a thorough review of the existing literature. A rigorous inclusion/exclusion protocol was then used to refine the list and shorten it to a sub-set of 27 indicators. An aggregate sustainability score was then computed based on those 27 indicators organized into four dimensions: environment, social, food security & nutrition and economic. The paper shows how the availability of data (or lack therefore) results in an unavoidable trade-off between number of indicators and number of countries, and highlights how optimization can be used to present the most robust metric possible given the existence of this trade-offs in the data space. The process results in the computation of a global sustainability map covering 97 countries and 20 indicators. The sustainability scores obtained for each country are made available over the entire range of indicators.

## Abstract (summary)

**Table:** 

Measurement(s)	food systems sustainability
Technology Type(s)	data transformation
Factor Type(s)	Country • Year
Sample Characteristic - Environment	climate system • cropland ecosystem • anthropogenic environment
Sample Characteristic - Location	Earth (planet)

Machine-accessible metadata file describing the reported data: 10.6084/m9.figshare.10011860

## Background & Summary

Addressing the question of the (un)sustainability of our food systems is critical as the world is bracing for hard-choice challenges and potentially massive trade-offs around issues related to food quality and food security in the coming decades^1,2^. Meeting increasing demand for nutritious food for a growing global population under climatic pressures, while mitigating associated environmental damages, is already a pressing challenge^1,3^. In 2016 the total number of chronically undernourished people was estimated to be around 815 million (more than one person out of ten)^1^. At the same time, the health consequences of the exponential increase in overweight and obese people are becoming another global burden^4,5^. Worldwide, those trends are correlated with a massive environmental ‘food print’ of the food production and distribution sectors^6,7^, coupled with patterns of food utilization characterized by concerning levels of waste and with supply chains that are increasingly homogenous and prone to crowding-out of smaller agri-food operators^8^.

While some conceptual and theoretical advances in defining food systems and their related indicators and metrics have shed light on these complex dynamics^9^, researchers and analysts are still struggling with one basic question: How can we define and empirically measure food systems’ *sustainability*? Attempts to address this question add insight^10,11^ but several conceptual or methodological challenges limit the overall utility of those efforts:

i. **Lack of representativeness**. Generally the list of countries included in such analyses is limited and often biased towards OECD or high income countries (for which data are usually more available than for lower income countries where national statistical systems are less effective). For instance the Sustainability map proposed by ref.^10^ covers only 67 countries –mainly high income countries;
ii. **Lack of conceptual clarity** on how the different dimensions of food system sustainability are constructed and delimited. While the most comprehensive of those studies do include series of indicators that cover four dimensions (social, environmental, nutrition and food security, and economy), it is not always clear how those indicators have been selected or can be rigorously measured. For instance ref.^11^ proposed a “resilience” dimension which they argue should be part of the sustainability assessment of food systems. The problem is that resilience is itself a latent variable (i.e. a variable that cannot be measure directly) and there is no particular reason why the index used by ref.^11^ (the ND-GAIN country index^12^) should be chosen over any other measure of resilience. In fact some even argue that there is currently no clear consensus on how to measure resilience, or whether resilience is really a dimension of (food system) sustainability^13^.
iii. **Replication and/or strong cross-correlation amongst indicators**. A detailed review of these different studies also reveals a lack of coherency about why certain indicators are included (or not) in the metrics. As a consequence, one often faces what could be termed a ‘shopping list’ syndrome, whereby a very long catalogue of indicators are proposed without clear justification about their inclusion. The consequence is that there is a very high risk of introducing some strong cross-correlation or replication in the metrics and, consequently, over-weighting artificially particular dimensions of the metric. For instance, in the nutrition dimension of ref.^10^, the authors have included both the Prevalence of stunting, and the Prevalence of wasting. While those indicators measure two slightly different aspects of undernutrition, it is well established that the trends of those two indicators are usually closely correlated at country level.
iv. **Sensitivity of the aggregated score** to the formula used to calculate the composite indicator. A point that is addressed only cursorily in related studies is the fact that the value of the overall scores that are computed is highly dependent on the formula used to aggregate all the different indicators included in the metric. Depending on the ways those different indicators are expected to interact, some formulas are more appropriate than others. A whole body of literature on composite indicators is available and should be used to guide our choice^14–16^.

The prevalence of these issues in existing approaches affects our ability to both assess and measure, and to compare food system sustainability in a robust and consistent manner. Yet, understanding the various dimensions of food systems’ sustainability and the degree to which food systems are (or are not) sustainable is critical in order to inform and support policy-makers in the design and implementation of adequate policy and interventions.

In this context, the objective of this paper is to build a *rigorous* metric of food system sustainability. By ‘rigorous’ we mean: (1) a metric that derives from a transparent and justified protocol, and (2) that maintains strict consistency with certain explicit quality parameters. In particular the metric should satisfy the four technical issues listed above. The details of this protocol are presented in the next sections of this paper.

## Methods

The methodology for this research explicitly addresses the technical issues described above. In this section, we present the different steps of the protocol implemented to achieve this consistent and coherent metric. The approach unfolds in two stages: (1) building the food system sustainability metric and populating it with the appropriate indicators; and (2) computing the sustainability score.

### Building the metric of food system sustainability

**Fig. 1:**
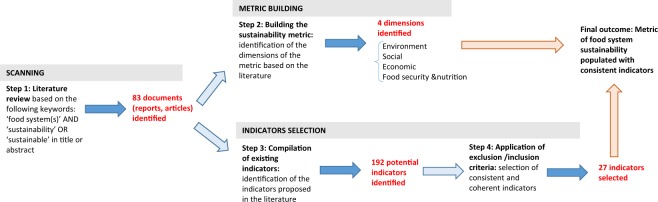
The four steps adopted to build and populate the food system sustainability metric.

We developed a four-step process with the objective to facilitate a consistent and reproducible approach to identifying indicators for food system sustainability. Those four steps are synthesized in Fig. 1 and discussed in detail below. The underlying motivation of this first stage was to establish a transparent and replicable protocol to build the metric and to identify relevant and usable indicators.

First, a series of keywords were used to identify peer-reviewed articles, documents and reports from expert groups and international development agencies that discuss indicators and metrics of food system sustainability (*step 1*). The key words used for this search were “food system(s)” AND (“sustainability” OR “sustainable”) found in the title or abstract of the document. The identification of the relevant documents was made through a systematic search through several multidisciplinary databases including Google scholar, JSTOR, and Scopus. The search for publications was limited to the period 2000–2018 given the relatively recent emergence and solidification of “food systems” as a defined research area. Eighty-three documents comprised the output of this step 1 search.

The review of those 83 document (*step 2*) indicates that four dimensions of sustainability appear to be almost universally acknowledged in the literature related to food systems: ecological, economic, social and food security & nutrition^5,17–19^. Though all four dimensions are arguably complex and compound in nature, two of the four (the environmental and food security & nutrition dimensions) are usually further decomposed into specified sub-dimensions. For environment, the five sub-dimensions most frequently proposed in the literature are: air, water, soils & land, biodiversity, and energy^3,9,20,21^. For food & nutrition, the sub-dimensions are: food security, food safety, food wastes & losses, and nutrition^22–24^. Altogether, those four dimensions and related nine sub-dimensions constitute the first two levels of the food system sustainability metric. These are presented in the first two columns of Table 1.

**Table 1:** Metric of food system sustainability.

Dimensions	Sub-dimensions	Categories
Environment	Air	▪ Quality
	Water	▪ Quality
		▪ Use
	Soils and land	▪ Quality
		▪ Use
	Biodiversity	▪ Crops
		▪ Wildlife (plants, animals)
	Energy	▪ Use
Economic		▪ Financial performance
		▪ Employment rate
		▪ Economic distribution
Social		▪ Gender/Equity
		▪ Inclusion (international)
		▪ Inclusion (national)
Food & Nutrition	Food Security	▪ Availability
		▪ Access (affordability)
		▪ (Physical) accessibility
		▪ Utilization – water
		▪ Utilization – energy
		▪ Stability (economic)
		▪ Stability (supply)
	Food Safety	▪ Safety
	Food Waste and Use	▪ Loss and waste
	Nutrition	▪ Diet
		▪ Undernutrition
		▪ Overweight & obesity
		▪ Hidden hunger (micro-nutrient deficiency)

A third level (termed categories) was then added to the metric. As with the higher levels of the metric, the choice of those categories derives from what is conventionally proposed in the literature. Thus, for food security the four traditional domains of food security were included, namely: availability, accessibility, utilization and stability^25,26^. For water, and soils and land, two categories (‘quality’ and ‘use’)^27,28^ were incorporated while for the social dimension, issues of equity, gender and inclusion appear to be widely accepted^8,29^. Less consensus exists on what should be the categories representing the economic sustainability. For the present study, we selected financial performance (creation of value added), employment rate, and economic inequality^1,30,31^. Finally, nutrition was broken into diet and the traditional components of the triple burden of malnutrition, namely: undernutrition, overweight & obesity, and hidden hunger (micronutrient deficiency)^25,32,33^. These different categories are detailed in the last right column in Table 1.

With the four dimensions of the food system sustainability metric identified, the next step consisted in “populating” each category with indicators. For this we compiled the list of indicators that were proposed in the 83 documents identified through the initial literature review. One hundred and ninety two (192) different indicators were identified, along with the dimension or sub-dimension and, sometimes, the category of the metric to which they were linked (*step 3*). For some of those categories, up to five or more indicators could be found in the literature; for others only one. Many of the identified indicators however displayed one or several of the conceptual and/or technical issues mentioned in the introduction. Several of them in particular, are indicators that are only collected in high-income countries and thus lack the global scope needed for this exercise. Others replicate or are strongly correlated with other indicators in the list.

The refining of the potential list of acceptable indicators was then done using a series of inclusion/exclusion criteria (*step 4*). Those inclusion/exclusion criteria were designed to address the abovementioned conceptual issues and allowed the “fit” of each indicator to be documented in a consistent and rigorous manner. Nine inclusion/exclusion criteria were considered as follows:

- **Cross correlation**. Were *excluded* indicators which are closely cross correlated to another indicator already considered in the list. For instance, “proportion of population under global poverty line” and “percentage of population living under the poverty threshold” are very closely correlated. We would only keep one of those two indicators.
- **Conceptual relevance**. Were *included* indicators that could clearly be related to one of the four dimensions of the metric, that is: ecological, economic, social and food and nutrition dimensions – see also composite indicator criterion below.
- **Global scale**. Were *included* only indicators for which a database which covers at least 70 countries is available.
- **Global validity**. Were *excluded* indicators that refer to processes that are specific to some specific regions of the world. For instance, “Percentage of agricultural land lost yearly to desertification” was excluded as desertification is a phenomenon that by definition can only occur in some specific regions of the world.
- **Time period**. Were *excluded* indicators for which the database had information only prior to the year 2000.
- **Latent variables**. Were *excluded* indicators that are based on latent variables. For instance, indicators of “resilience” or “economic vulnerability” were excluded as there is no agreed measure/unit of resilience or economic vulnerability.
- **Clear methodology**. Were *excluded* indicators for which the methodology used to construct the database was not clearly detailed in the original database.
- **Single dimension indicators**. Were *excluded* indicators based on ‘multi-dimension’ indices that fall into two different dimensions of the metric. For instance, the ratio “natural capital used/GDP” which is sometimes proposed in the literature as an indicator of sustainability would not be included as it clearly lies at the interface between the environmental and economic dimensions.
- **Comparability**. Were *excluded* (or amended) indicators that were based on absolute numbers that do not allow for comparison between countries – for instance the total number of km of paved roads would not be included. Instead the road density was considered, that is, the total number of km of paved road per 100 square km of land area.

Applying the inclusion/exclusion criteria to the pool of 192 indicators allowed us to short-list a subset of 27 indicators. We present this final set of 27 indicators in Table 2, along with the dimensions of the metric to which they are related. Also indicated in the subsequent columns are several characteristics of these indicators, including the period for which the data are available, their sources, and the number of countries for which the data are currently recorded. Those characteristics will be discussed in greater detail below. Amongst those 27 indicators, seven are of environmental nature, three economic, three social, and 14 food & nutrition.

**Table 2:** The metric of food system sustainability and the 27 indicators which were selected to populate the metric.

Dimension	Sub-dimension	Category	Indicators^(a)^	SR^(b)^	DP^(c)^	Source	Period	Nber countries^(d)^
Environment	Air	Quality	Greenhouse gas emissions in total agriculture (gigagrams)	−	C	FAO	2000–2010	222
	Water	Quality	Water pH	−	C	GEMStat water quality database	1965–2016	74
		Use	Agricultural water withdrawal as percentage of total renewable water (%)	−	P	FAO	2000–2016	174
	Soil and land	Quality	Soil carbon content (as percentage in weight)	+	C	FAO	2008	202
		Use	Agricultural land as % of arable land	−	C	FAO	2000–2014	217
	Biodiversity	Wildlife (plants, animals)	Benefits of biodiversity index (0 = no biodiversity potential to 100 = maximum)	+	C	The Global Environment Facility	2008	192
			Crop diversity (Calories diversity measured by Shannon index)	+	C	Khoury et al., 2016	2009–2011	177
	Energy	Use	Agriculture and forestry energy use as % of total	−	P	FAO	2000–2009	113
Economic		Financial performance	Agriculture value-added per worker (constant 2010 US$)	+	P	The World Bank	2000–2015	181
		Employment rate	Agriculture under-employment (%)	−	P	International Labour organization - UN	2000–2014	72
		Economic distribution	Gini index for land distribution & tendency	−	P	GRAIN organization	1989–2013	86
Social		Gender equity	Labor force participation rate, female (% of female population ages 15+)	+	P	The World Bank	2000–2016	184
		Inclusion	Predominant fair trade organizations and producers	+	P	Fairtrade International	2016	160
			Employment in agriculture (% of total employment)	+	P	The World Bank	2008–2017	149
Food and Nutrition	Food Security	Availability	Per capita food available for human consumption (kcal/capita/day)	+	C	Dupon_GFSI source FAO	2016	113
		Access	Food consumption as share of total income (% of total household expenditure)	−	C	Dupon_GFSI_National Accounts; United Nations	2016	113
			Estimated travel time to the nearest city of 50,000 or more people (Hours travel from a city)	−	C	European Commission	2015	245
		Utilization	Access to improved water resource (% of total population)	+	C	FAO	2000–2014	198
			Access to electricity (%)	+	C	The World Bank	2000–2014	211
		Stability	Price volatility index	−	C	FAO monthly CPI	2011–2017	194
			Per capita food supply variability (kcal/capita/day)	−	C	FAO	2000–2011	162
	Food Safety		Burden of foodborne illness (number of cases)	−	C	WHO	2010	194
	Food waste and Use		Food loss as % of total food produced	−	C	Dupon_GFSI source FAO	2016	113
	Nutrition	Diet	Diet diversification	+	C	FAO	2001–2010	165
		Undernutrition	Stunting, children aged < 5 years stunted (%)	−	C	WHO	2000–2014	129
		Overweight & obesity	Prevalence of obesity (% of the population, over 18 y of age	−	C	WHO	2000–2014	191
		Hidden hunger	Serum retinol deficiency	−	C	WHO	1995–2005	193

Notes: ^(a)^the details of the indicators’ definitions are available from the metadata table provided in the Harvard Dataverse record^36^; ^(b)^SR: Sign of the Relationship: the expected sign of the relationship between the indicators and the level of sustainability, e.g. a + sign would refer to situation where in theory we would expect that the higher the indicator the higher the level of sustainability; ^(c)^DP: Degree of Proxy: the extent to which the proposed indicator captures the process in a holistic manner (noted C), or just part of it – in that case it means the indicator acts only as a proxy (P) for the whole system–see text for details; ^(d)^Number of countries based on the ISO standard “country code” list, which includes 249 countries, territories and areas of geographical interest.

### Computation of the sustainability score

The second stage in the protocol was the computation of the aggregate score based on the 27 indicators. First a Box Cox transformation was applied to the most skewed indicators –i.e. those with a |Skew(x) − 0| > 2 – to improve the normality of distribution and avoid potential issues related to heteroskedastic dataset distributions. Once those indicators were transformed, the individual indicators were normalized using a standard (rescaling) min-max transformation with a [0, 1] range.

We then identified the most appropriate approach to compute the aggregate score. For this, we referred to the literature on composite indicators and multi-criteria decision analysis. This literature is well established and offers two relevant rules with respect to composite indices^16,34,35^.

**Fig. 2:**
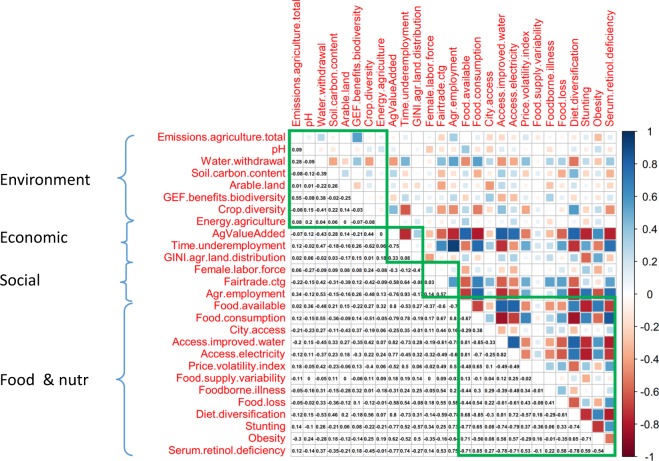
Spearman-correlation matrix of the 27 indicators included in the food system sustainability metric. High positive correlations are indicated in dark blue, while high negative correlations are showed in dark red. The diagram shows that the environmental and social dimensions are characterized by low internal cross-correlations, while the economic and food & nutrition dimensions display a larger number of high positive and/or negative cross-correlations.

- if the different dimensions of the metric are expected to be compensatory (that is, if one or several dimension(s) can be substituted by other(s)), then a simple arithmetic mean is sufficient to calculate the aggregate score of the index. If, on the contrary, the dimensions are supposed/expected to be non-compensatory, then other approaches to aggregation should be considered. Strict non-compensatory aggregation methods like multi-criteria analysis can be used in order to control absolutely for highly unequal values, or a geometric mean can be used to reduce the effects of highly unequal values on the aggregate score. In our case, we assumed to be minimal compensatory effects between the four dimensions of the sustainability metric (environmental, economic, social, and food & nutrition) as we did not expect that a particular dimension could be fully substituted by any other. We therefore applied a geometric mean between the four dimensions of the aggregate score;
- if the different elements/variables within one dimension are highly correlated with each other, an arithmetic formula should be used within that particular dimension; if on the contrary the degree of cross-correlation between the variables appears to be low, then a geometric mean should be used. The cross-correlation matrix of the 27 indicators was computed and the results are shown in Fig. 2. The diagram shows that the environmental and social dimensions of the metric are characterized by low internal cross-correlations – thus geometric means were used for those two dimensions – while the economic and food & nutrition dimensions display several high positive and/or negative cross-correlations; arithmetic means were therefore used for those two other dimensions.

**Fig. 3:**
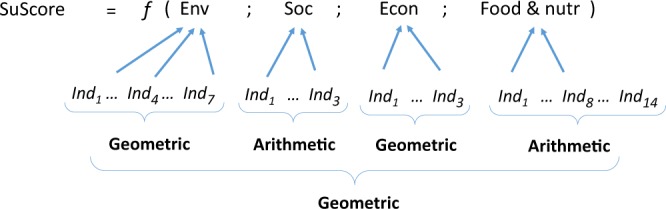
Formula used for the computation of the aggregate sustainability score.

Applying the two rules presented above, the overall formula used for the aggregate score is shown in Fig. 3, where SuScore is the aggregate score, *Ind1* to *Ind14* represent the 27 indicators associated with the four dimensions, which themselves are represented by the symbols Env, Soc, Econ, and Food & nutr. ‘Geometric’ and ‘Arithmetic’ in the formula indicate the types of mean used within and between the dimensions of the metric to aggregate the indicators.

## Data Records

In this section we discuss in greater detail the 27 indicators presented in Table 2, providing an overview of their origins, the repository where they are available, and their characteristics in relation to their assumed contribution to the food system sustainability metric. We then discuss a critical issue in relation to the construction of global composite maps, that is, the trade-offs between the number of countries and the number of indicators that can be included in those maps.

### Food system sustainability datasets – an overview

The 27 indicators that had been short-listed through steps 1–4 are listed in the fourth column (labelled ‘indicators’) of Table 2. Their detailed definition as well as where they can be retrieved is provided in the Harvard Dataverse database “Sustainable food systems global index”^36^. For the Environmental dimension, seven indicators that satisfied the inclusion/exclusion criteria had been identified from the literature. Those cover five sub-dimensions of the environmental dimension: the quality of air, the quality and use of water, the quality and use of soils and land, the level of wildlife biodiversity and crop diversity, and the use of energy. For the Economic dimension of the sustainability metric, three indicators that satisfy all the inclusion/exclusion criteria were identified from the literature. They cover the financial performance, level of employment, and economic distribution of the wealth generated by the food system. Likewise, for the Social dimension, only three indicators satisfy all the inclusion/exclusion criteria. They cover the gender/equity and the degree of inclusion of the system (both international and national levels). Finally for the Food & Nutrition dimension, a richer set of indicators is available from the literature and 14 indicators satisfying the inclusion/exclusion criteria were identified. They cover the four standard elements of food security (availability, access, utilization, and stability), plus food safety, food waste and use, and the four conventional dimensions of nutrition, that is, diet quality, undernutrition, overweight & obesity, and micronutrient deficiency.

The next column labelled “SR” in Table 2 indicates the expected sign of the relationship between the individual indicators and the resulting level of sustainability. A positive (+) sign would refer to situations where a positive relation is theoretically expected between the indicator under consideration and the overall level of sustainability. For instance, it is reasonable to assume that the higher the level of carbon in the soil, the higher the quality of the soil and the higher the sustainability of the system; likewise the higher the diet diversity index, the better the quality of the diet and the higher the sustainability of the food system. Those indicators are therefore associated with + signs. In contrast a negative (−) sign indicates a situation where a high value of the indicator is expected to be associated with a low level of sustainability of the food system. Examples include price volatility index or prevalence of obesity. Overall the SR column indicates that all indicators selected have an expected monotonic relationship with the sustainability of the system, which is an important property as it reduces the risk of complications that non-monotonic relations would introduce for the interpretation of the global index. Note that in that regard the data of the water pH (capturing the water quality sub-dimension) has been transformed using the absolute value of the difference between the actual pH value and 7 (reference value) so that the SR sign for this specific set of data is also monotonous and negative.

The next column, labelled “DP”, indicates the Degree of Proxy with respect to food system, that is, the extent to which the indicators included in the metric capture the process they are expected to measure *in a comprehensive manner*, or whether they only capture part of it. For instance the indicator used to reflect the degree of gender equity is the index “Female employment rate *in agriculture*” currently compiled by the World Bank, based on national statistics^37^. This index captures gender equity *in the agriculture sector only*. In its current form, it does not say, therefore, anything about the situation in the other sectors of the food system, such as processing, retailing or distribution. It means the indicator currently available for gender equity is only a proxy for the whole food system. As such it is associated with a “P” in the column DP in Table 2 (“P” for partial). In contrast the level of biodiversity, which by definition concerns the pre-production sector, is captured adequately in the metric by the biodiversity index as computed by the Global Environment Facility^38^. This indicator can therefore be considered as covering comprehensively the part of the system concerned with this specific issue. It is therefore associated with a “C” (for ‘comprehensive’) in the DP column.

The overall proportion of P’s and C’s in the column DP provides us with a qualitative indication of the level of ‘coverage’ provided by the indicators that were found in the literature. As far as Food & nutrition is concerned the situation is relatively satisfactory – since all the indicators are characterised as C-indicators. The situation of the Environmental dimension is more mixed, with 6 Cs- and 2 Ps. On the other hand, the Social and Economic dimensions of the metric are, at the present time, only partially captured. Both dimensions are represented only by P-indicators. This partial coverage is mainly due to the fact that all the indicators available at the present time at a global level are indicators that capture social or economic aspects *of the agriculture sector*; they do not include information related to the other sectors of the system, such as transport, distribution, transformation that are also part of food systems.

The next column in Table 2 indicates the original sources from which the indicators were retrieved. The large majority of them come from UN agencies – in particular the Food and Agriculture Organization – which generally collect information/data from their member countries’ national statistics. Exceptions to this are (i) the data related to the number of fair trade organizations and producers, which was compiled by the NGO Fairtrade International, (ii) the estimated travel time to the nearest city, made available by the European Commission, (iii) the Price volatility index computed by the International Center for Tropical Agriculture (CIAT), and (iv) the Crop diversity index^39^. In those last two cases however (price volatility and crop diversity) the initial datasets used to compute those higher level indicators were initially derived from UN-FAO datasets.

Last on the right-hand side of Table 2 are the columns that indicate the time-period and the number of countries for which these different datasets are available. The columns show that all datasets cover the period 2000–2017 of interest to us, and that (at the present time) the dataset with the lowest number of countries is the rate of under-employment in the agriculture dataset (72 countries), while the indicator with the largest number of countries and territories is the travel time (currently computed for 245 countries and territories).

### Countries – optimizing indicator coverage

**Fig. 4:**
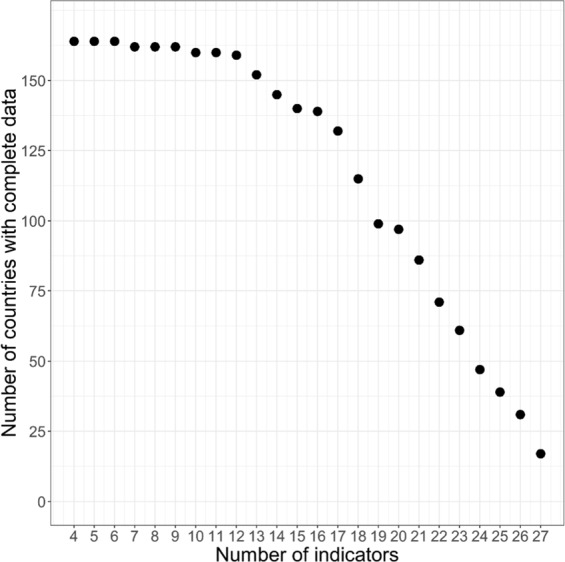
Trade-off ‘frontier’ between the number of countries for which the datasets of indicators are complete and the number of indicators included in the metric. The frontier shows that the larger the number of indicators considered, the smaller the number of countries for which those indicator datasets are complete, and vice versa.

**Fig. 5:**
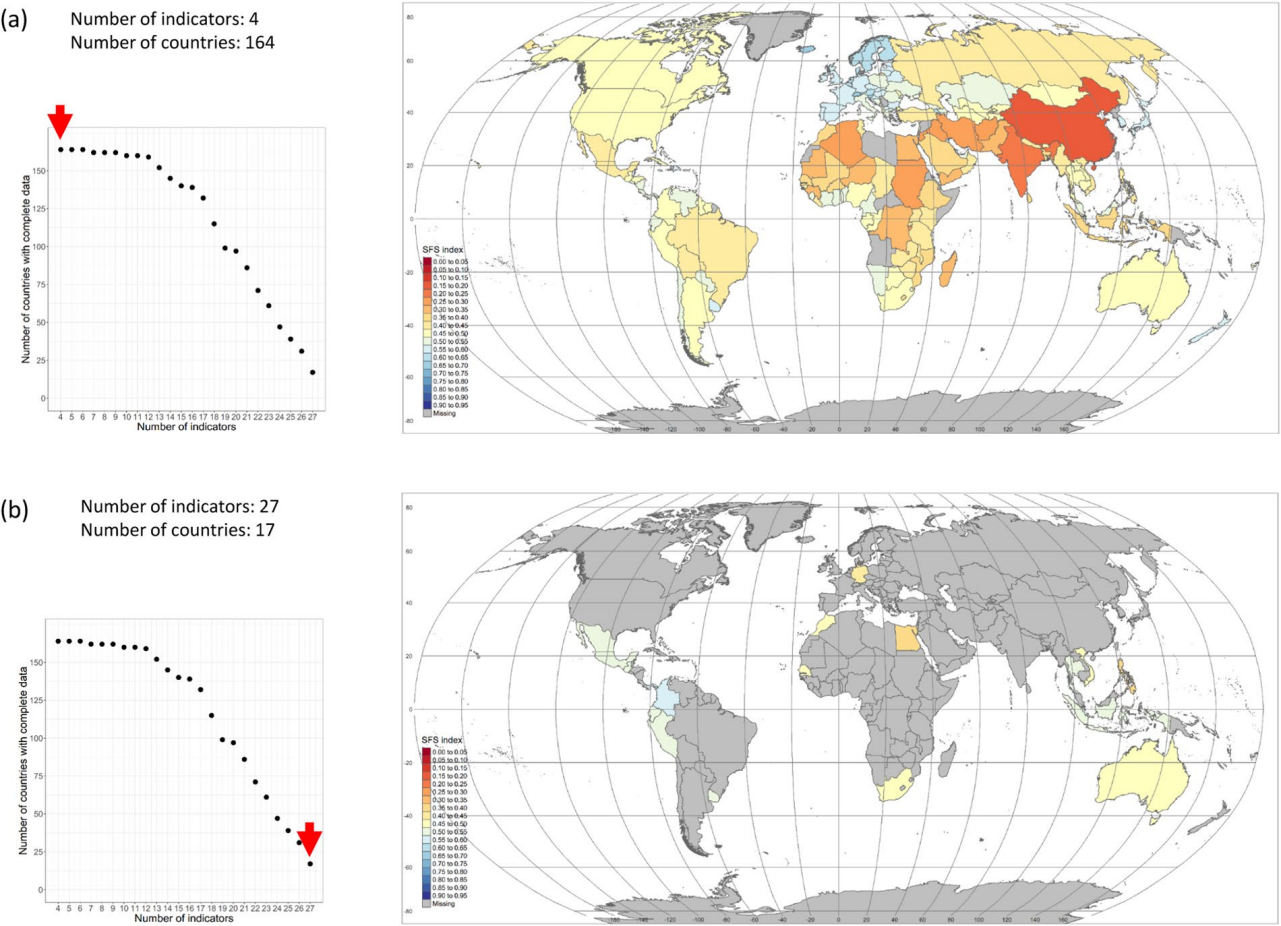
Sustainability maps for extreme cases. (**a**) Top graph - map generated for the maximum number of countries (164) for which at least one indicator exists in each of the four dimensions of the metric. (**b**) Bottom graph - map generated for the maximum number of indicators (27). Only 16 countries have complete dataset of all 27 indicators. The smaller diagrams on the left-hand side of the two maps display the trade-off frontier between number of countries with complete datasets and number of indicators. The red arrows indicate the combinations countries-indicators included in the two maps: 164 countries – 4 indicators for (**a**) and 16 countries – 27 indicators for (**b**).

One critical issue, albeit rarely discussed in papers dealing with the construction of global metrics, is the trade-off that exists between the number of countries included in the analysis and the number of indicators used to build the metric. It is important to understand that each indicator in the metric is available for a specific subset of countries and that those countries are not always the same across indicators. For instance, although the FAO per capita food supply variability index and the Predominant fair trade organizations and producers dataset constructed by Fairtrade International both cover a very similar number of countries (162 and 160 respectively), the actual number of countries that is common to the two datasets is only 118. The implication is that it is not possible to maximize the two dimensions of the metric (countries *and* indicators) at the same time and a choice (trade-off) has to be made. In the present case for instance, the maximum number of countries for which at least one indicator in each of the four dimensions of the metric is available, is 164. On the other end, if we want to retain the 27 indicators initially identified, only 16 countries with complete datasets for all 27 indicators can be found. This situation creates a ‘trade-off frontier’ – displayed on Fig. 4. In parallel Fig. 5 shows the two extremity scenarios mentioned above: the maximum number of countries (164) for which at least one indicator is available in each of the four dimensions of the metric (Fig. 5a); and the set of 16 countries for which data are available for all 27 indicators (Fig. 5b).

## Technical Validation

The existence of trade-offs between countries and indicators has technical implications for the final computation of the sustainability score. In particular the three issues encountered during the construction of the aggregate score were:

(i) Stability of the aggregate score
(ii) Choice of the optimal combination of countries and indicators
(iii) Sensitivity of the aggregate score to the number of indicators included in each dimension of the metric

### Stability of the composite score

**Fig. 6:**
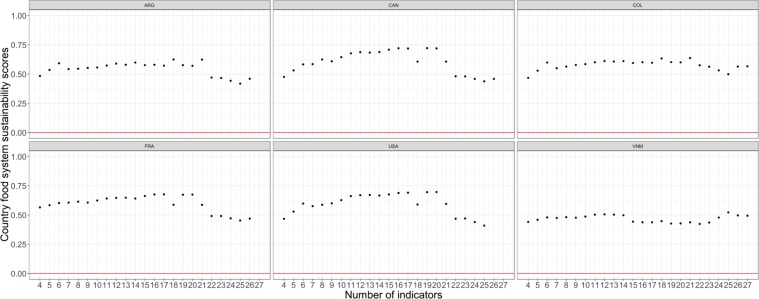
The six diagrammes illustrate the sustainability score stability issue. Each diagramme shows the sustainability score computed along the trade-off frontier for one specific country (ARG = Argentina; CAN = Canada; COL = Colombia; FRA = France; USA = United States of America; VNM = Vietnam). The figure shows how the country individual score fluctuates heavily when the number of indicators included in the metric goes from 4 to 27, suggesting an instability issue.

The first technical complication we faced while computing the composite score was a stability issue. Figure 6 illustrates this issues for 6 countries (Argentina, Canada, Colombia, USA, France and Vietnam) taken as examples. The figures shows that when the number of indicators included in the metric goes from 4 to 27, the individual country’s aggregate score does fluctuate, sometimes substantially. This instability is obviously an issue as it means that the countries’ scores and their subsequent rankings will vary heavily depending on the number of indicators included in the metric. The reason for this instability relates to the fact that –as explained above- the sets of countries for which indicators’ datasets are complete are not the same from one indicator to the next along the trade-off frontier. It means that when the indicator number increases from, say, 10 to 11, the set of countries that are included in the metric for 11 indicators is different from the countries which were included in the metric when only 10 indicators were considered. The different set of aggregations naturally result in different composite scores.

**Fig. 7:**
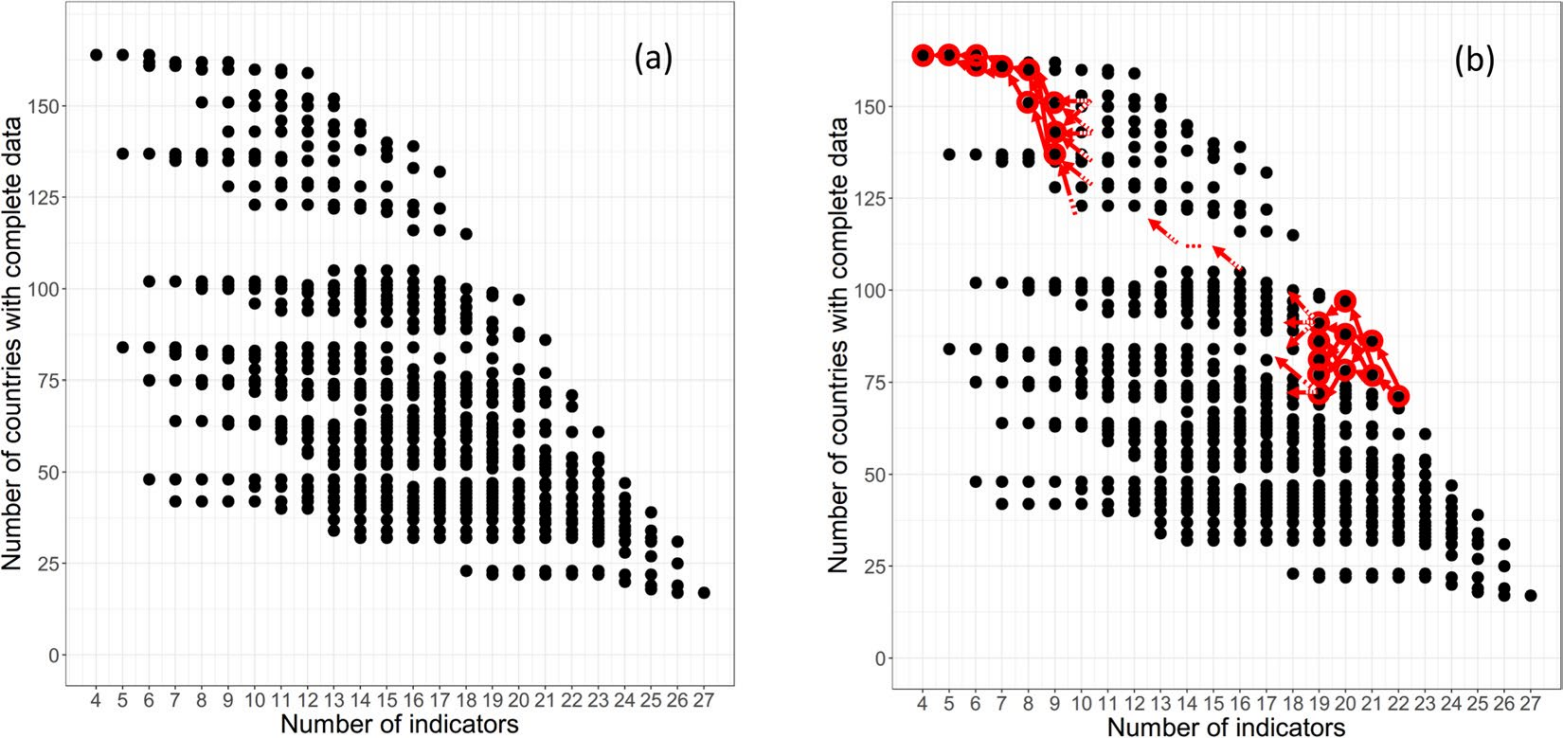
Map of countries – indicators combinations. (**a**) Left graph The 882 possible sets of combinations of countries and indicators that were created by combining the 164 countries and the 27 indicators. The points on the most outward part are those constituting the trade-off frontier. (**b**) Right graph - an illustration of the backward selection process, starting from the combination: 71 countries – 22 indicators. See text for details.

To manage this composite score instability issue, we needed to develop a calculation procedure that avoids the constant ‘reshuffling’ of countries between each level of indicators. For this we first mapped the entire sets of possible combinations of countries-indicators. Eight hundred and eighty two (882) combinations were thus identified (those are shown on Fig. 7a). Using this ‘map’ of possible combinations, we then applied a backward identification process to isolate the countries-indicators combinations for which the sets of countries would remain as similar as possible from one level of indicators to the next: starting from a given combination on the trade-off frontier we used dynamic programming (backward chaining) to identify the successions of indicators which include the same set of countries (plus the additional one(s) associated to the next lower level of indicators), moving backward (or leftward) through the map of possible combinations, until this process takes us back to the initial combination of 164 countries and 4 indicators. This backward chaining process is illustrated in a schematic way in Fig. 7b for the combination 71 countries – 22 indicators.

**Fig. 8:**
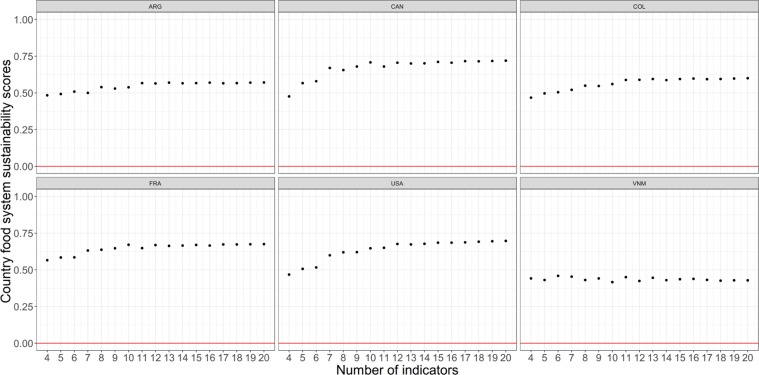
Computation of the sustainability score for the six countries that were initially shown in Fig. 6 after the backward selection process was applied (ARG = Argentina; CAN = Canada; COL = Colombia; FRA = France; USA = United States of America; VNM = Vietnam). The figure shows how the backward selection process was successful at stabilizing the countries’ individual sustainability scores.

The backward selection process was successful at reducing the instability in the score computation. Figure 8 shows the resulting sustainability scores for the six countries that were initially shown in Fig. 6. The six countries now display sustainability scores that are far more stable across the range of indicators than the initial ones (compare Fig. 8 with Fig. 6).

### Choice of the optimal combination

The second major challenge we faced in constructing the global sustainability map was to identify which combination of countries and indicators was the ‘optimal’ one. The essence of the question was: should we try to retain 20, 21, 22 or more indicators, or shall we try to keep 110, 120 or more countries? Fig. 5a,b had already highlighted the problem: maximizing the number of indicators included in the metric would mean reducing the number of countries for which the dataset is complete, while maximizing the number of countries would leave us with a reduced number of indicators. This optimal combination can be found along the trade-off frontier, but how could we decide which combination is the ‘right’ one?

To address this question we considered three decision criteria:

(i) the number of countries that are dropped out of the metric when the number of indicators is increased by 1 -we knew that each time a new indicator is added to the metric, the number of countries for which the full set of indicators is available decreases – we therefore computed the drop in this number of countries and used it as the first decision criterion;
(ii) the variability in the countries’ sustainability scores - we knew that changing the number of indicators included in the metric still slightly affects the sustainability scores of the countries remaining in the metric even after applying the backward process described above (scores as shown in Fig. 8 are stabilized but clearly not perfectly constant across the range of indicators) – we therefore estimated this variability by computing the standard deviation of the scores’ aggregated mean and used it as the second decision criterion;
(iii) the level of rank-shifting experienced by those countries - we knew that each time a new indicator is included in the metric, not only the value of the countries’ score fluctuates (point (ii) above), but the subsequent ranking of these countries may also change – we therefore computed this rank-shifting and used it as our third decision criterion.

The objective was then to determine which particular countries-indicators combination displays the lowest combined value when the three criteria are considered together. Using a simple minimal function (seeking for the lowest value of the aggregated normalized criteria) we were able to identify that the combination 20 indicators – 97 countries was the optimal combination (for the current dataset available).

**Fig. 9:**
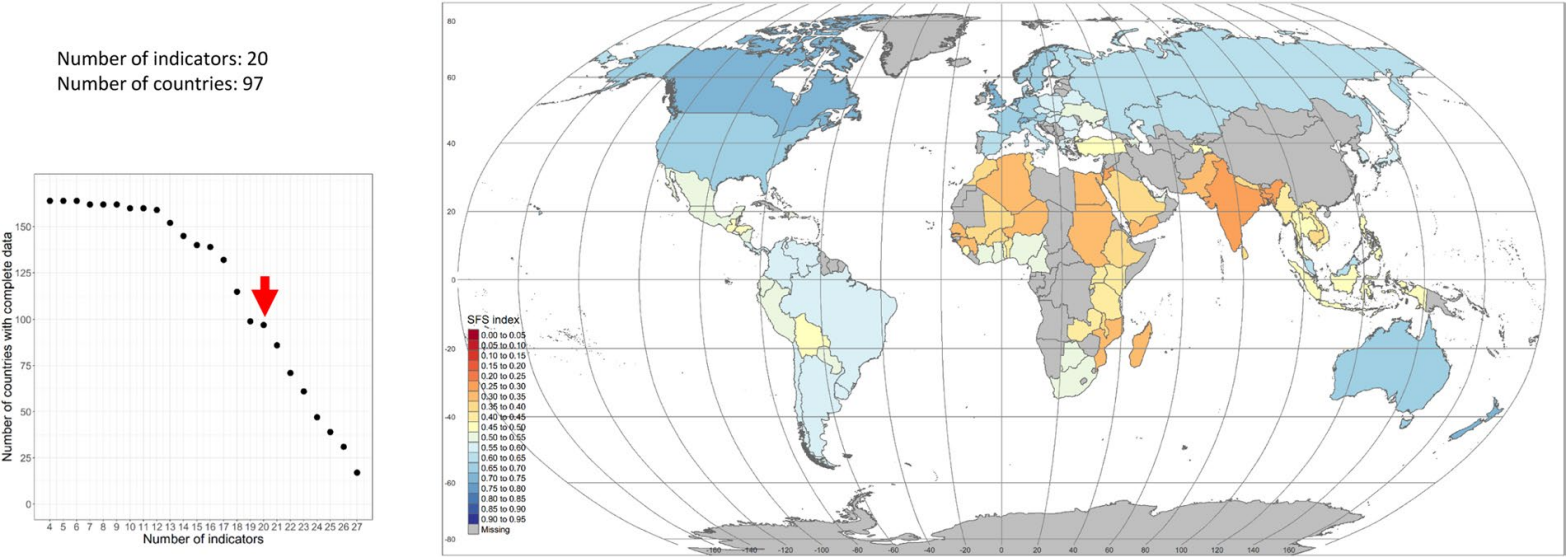
Global map of food system sustainability obtained for 20 indicators and 97 countries. The list of indicators used to build the map is provided in Table 3. Country individual scores are provided in the metadata table provided in the Harvard Dataverse record^36^.

The food systems’ sustainability global map which results from applying the series of steps described above is shown in Fig. 9 for 2017 and the 20 indicators that are included in the metric are listed in Table 3. Six of them belongs to the Environment dimension, one to the Social dimension, one to the Economic dimension, and 12 to the Food & nutrition dimension.

**Table 3:** The list of the 20 indicators included in the final global sustainability map, along with their dimensions.

Environment	Economic
• Greenhouse gas emissions • Agricultural water withdrawal • Soil carbon content • Agricultural land as % of arable land • Benefits of biodiversity index • Crop diversity	• Agriculture value-added per worker
**Social**	**Food & nutrition**
• Labor force participation rate, female (% of female population ages 15+)	• Per capita food available for human consumption • Food consumption as share of total income • Estimated travel time to the nearest city of 50,000 or more people • Access to improved water resource • Access to electricity • Price volatility index • Per capita food supply variability • Burden of foodborne illness • Food loss as % of total food produced • Diet diversification • Prevalence of obesity • Nutrient deficiency

### Sensitivity of the aggregate score to the number of indicators included in each dimension of the metric

The third potential issue relates to the unequal number of indicators included in the different dimensions of the metric (see Table 3) and the possible implication that this unequal number has for the computed aggregate score. The question is whether the composite score is more sensitive to changes that occur in an indicator when that indicator is the only one currently included in a specific dimension (the case of the Social and Economic dimensions) than when the changes occur in an indicator included in a dimension that contains several other indicators (Environment and Food & nutrition).

This issue of unequal numbers of indicators in composite indexes is not new. For instance the Multidimensional Poverty Index developed at OPHI-Oxford^40^ or the related UNDP Human Development Index (HDI) are two famous examples of this situation (the MPI for instance is a 3-dimension index with 2/2/6 indicators in its respective dimensions). Those examples demonstrate that having different numbers of indicators between dimensions is acceptable (and accepted in the larger community) and does not prevent both academics and decision-makers from relying on those aggregate indexes for their analyses. Nevertheless, it is important to evaluate the extent to which this issue affects the food system sustainability score.

We therefore conducted a complementary sensitivity analysis to try to “quantify” this potential bias. For this, we compared the percentage changes that would be observed in the aggregate sustainability score under various scenarios. The results are presented in Table 4. They show that changes in the indicators included in the economic or in the social dimensions of the sustainability score have generally larger effects on the aggregated score than comparable changes in one of the indicators included in the Environmental and in the Food security & nutrition dimensions. But the results of the analysis also show that those changes in the aggregate score are relatively small. For instance a scenario with a substantial change in the economic indicators (whereby the value of the economic indicators is increased by 30% for 20 countries –out of the 97) results in only a 3% change in the aggregated score.

**Table 4:** Sensitivity analysis. The values reported in the table are percentage changes in the aggregate score following two scenarios: (i) “10-countries” scenario = the individual values of one indicator in each dimension of the metric is increased by 10%, 20%, and 30% for 10 countries (chosen randomly) amongst the 97 initial countries; (ii) “20-countries” scenario = the values of one indicator in each dimension was increased by 10%, 20%, and 30% for 20 countries (chosen randomly).

scenarios	10 countries			
Rate of change	environment	economic	social	Food&nutr
10%	−0.052%	0.807%	0.366%	−0.010%
20%	−0.113%	1.212%	0.697%	−0.021%
30%	−0.191%	1.535%	0.999%	−0.031%
	**20 countries**			
	**environment**	**economic**	**social**	**Food&nutr**
10%	−0.090%	1.946%	0.359%	−0.024%
20%	−0.194%	2.753%	0.498%	−0.048%
30%	−0.324%	3.070%	0.607%	−0.072%

Indirectly, this result also confirms that the computation approach we used (presented in the ‘Computation of the sustainability score’ section above) is relatively robust and limits the effects of those uneven dimensions, though it does not eliminate them totally.

## Usage Notes

The quality of any composite score as well as the soundness and utility of the messages conveyed depend not only on the methodology used in its construction but also on the quality of the underlying conceptual framework and data. A composite score based on convenience, availability, or loose theoretical basis, or on datasets containing large measurement errors, can lead to disputable policy messages and potentially maladaptive policy responses.

In this paper, we presented the first global map of food systems sustainability based on a metric that follows a completely transparent, reproducible, and rigorous protocol, and that maintains strict consistency with openly expressed quality parameters. The choice of the metric dimensions as well as the selection of the indicators included in that metric reflected the common understanding of this concept in the current literature. Our responsibility has simply been to apply a rigorous inclusion/exclusion protocol to trim down a long list of more than 190 proposed indicators to a shorter set of indicators. The outcome of this was the elimination of possible replications/cross-correlations, and greater assurance that the short-listed indicators have been generated or measured through a clear and well-established methodology. In this way, the individual indicators are unambiguously associated to only one dimension of the metric, and correspond to datasets for which data exist and are available for a large number of countries.

The final metric (97 countries and 20 indicators) that results from this rigorous process represents the best possible trade-off between the number of indicators and the number of countries, given (a) the current availability of data at the global level and (b) our initial objective to capture the four dimensions of sustainability. This specific combination is not of course the only possible way to interpret the data. Individual researchers who may have specific geographical interests or different priorities in terms of richness of indicators, number of countries to be considered, or focus on particular dimensions of sustainability, may decide to use a larger (or smaller) subset of the indicators or countries that are available through the dataset associated with this article^36^. It would be important to stress however that in that case the countries’ scores and ranks obtained with a different set of indicators will not be directly comparable to the current values as presented in Fig. 9, and should therefore be appropriately interpreted.

One of the other potential concerns in the use of the current score is the unequal number of indicators identified across the different dimensions of the metrics. In particular the small number of indicators related to both the economic and social dimensions (one and one respectively –cf. Table 3) could be seen as an important limitation of this work. While this number can be increased by considering a smaller number of countries within the metric, the fundamental issues is the lack of existing data reflecting the social or economic dynamics of national level food systems^24^. In total only six indicators satisfying all the inclusion/exclusion criteria across these two dimensions were identified in open access databases. Furthermore the presence of “P” symbols in Table 2’s DP column for the entire subset of social and economic indicators signals that the indicators that were identified are in fact only proxies for the processes they are expecting to represent. In other words the construction of this present metric also highlights the urgent need for governments and other key stakeholders (donors, international development agencies, etc.) to invest in more comprehensive monitoring of food systems -in particular in relation to sectors such as transformation, transport, retail and distribution, for which data are still missing, including in some high income level countries.

**Fig. 10:**
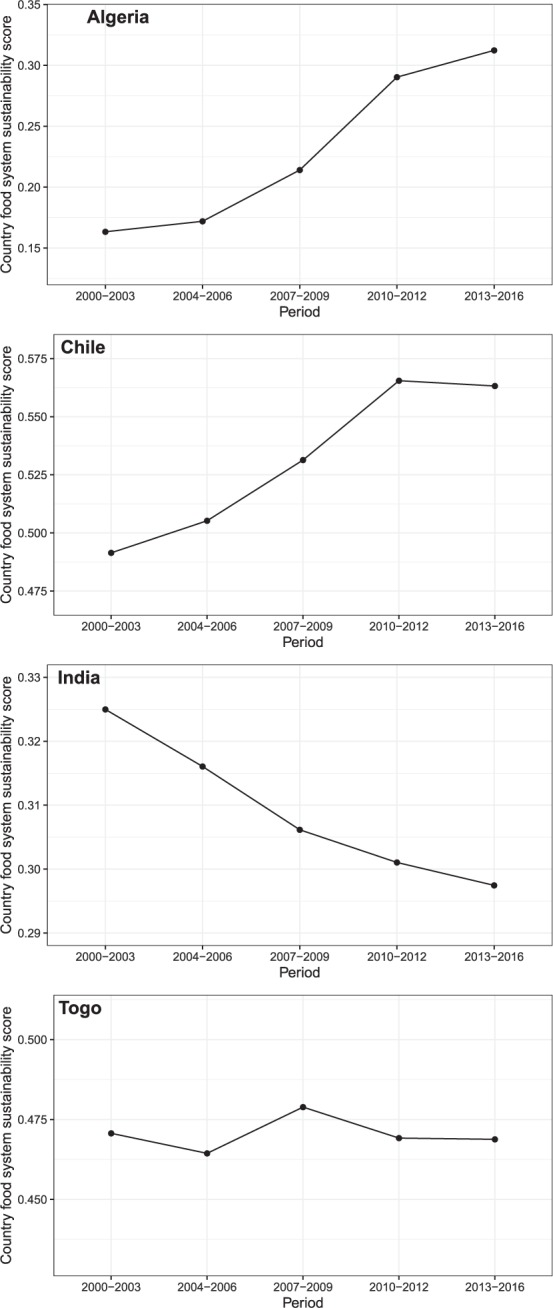
Change over time in country’s food system sustainability scores illustrated through the case of Algeria, Chile, India, and Togo. Changes computed for 5 sub-periods: 2000–2003; 2004–2006; 2007–2009; 2010–2012; 2013–2016. The graphs show that over the period 2000–2016, Algeria and Chile have shown a substantial improvement in the sustainability of their food systems, while Togo’s score has remained relatively constant and India’s one declined.

Finally it is important to acknowledge that because of the multi-dimensional nature of the sustainability score, there is no ‘natural’ or ‘theoretical’ threshold above which a country can be said to be sustainable. As such, the virtue of this work is not in helping people or experts categorize or label countries as “sustainable” or “unsustainable” in relation to the status of their food systems. Instead the value of the metric and its scores lies in the possibility they offer to compare levels of food systems’ sustainability, not just between countries, but also over time for a given country or a group of countries. Figure 10 represents for instance the changes observed for four different countries: Algeria, Chile, India and Togo over the period 2000–2016. This possibility to document change over time can provide an extremely useful tool for monitoring countries’ progress toward their own objectives of (food systems)’ sustainability or to assess the effect of particular drivers^41^, as well as helping to identify the most effective policy strategies, given specific national contexts.
